# Segregation of Incomplete Achromatopsia and Alopecia Due to *PDE6H* and *LPAR6* Variants in a Consanguineous Family from Pakistan

**DOI:** 10.3390/genes7080041

**Published:** 2016-07-27

**Authors:** Christeen Ramane J. Pedurupillay, Erlend Christoffer Sommer Landsend, Magnus Dehli Vigeland, Muhammad Ansar, Eirik Frengen, Doriana Misceo, Petter Strømme

**Affiliations:** 1 Department of Medical Genetics, Oslo University Hospital, Oslo 0450, Norway; p.j.c.ramane@medisin.uio.no (C.R.J.P.); magnusdv@medisin.uio.no (M.D.V.); eirik.frengen@medisin.uio.no (E.F.); doriana.misceo@medisin.uio.no (D.M.); 2 Faculty of Medicine, University of Oslo, Oslo 0316, Norway; 3 Department of Ophthalmology, Oslo University Hospital, Oslo 0450, Norway; erllan@ous-hf.no; 4 Department of Genetic Medicine and Development, University of Geneva, Geneva 1211, Switzerland; Muhammad.Ansar@unige.ch; 5 Women and Children’s Division, Department of Clinical Neurosciences for Children, Oslo University Hospital, Oslo 0450, Norway

**Keywords:** achromatopsia, alopecia, phosphodiesterase (PDE), *PDE6H*, *LPAR6*

## Abstract

We report on two brothers with visual impairment, and non-syndromic alopecia in the elder proband. The parents were first-degree Pakistani cousins. Whole exome sequencing of the elder brother and parents, followed by Sanger sequencing of all four family members, led to the identification of the variants responsible for the two phenotypes. One variant was a homozygous nonsense variant in the inhibitory subunit of the cone-specific cGMP phosphodiesterase gene, *PDE6H*:c.35C>G (p.Ser12*). PDE6H is expressed in the cones of the retina, which are involved in perception of color vision. This is the second report of a homozygous *PDE6H*:c.35C>G variant causing incomplete achromatopsia (OMIM 610024), thus strongly supporting the hypothesis that loss-of-function variants in *PDE6H* cause this visual deficiency phenotype. The second variant was a homozygous missense substitution in the lysophosphatidic acid receptor 6, *LPAR6*:c.188A>T (p.Asp63Val). LPAR6 acts as a G-protein-coupled receptor involved in hair growth. Biallelic loss-of-function variants in *LPAR6* cause hypotrichosis type 8 (OMIM 278150), with or without woolly hair, a form of non-syndromic alopecia. Biallelic *LPAR6*:c.188A>T was previously described in five families from Pakistan.

## 1. Introduction

Consanguineous marriage, a union between two individuals who are related as second degree cousins or closer, is rooted in cultural and socio-economic factors and is estimated to affect one fifth of the world population [1,2]. Progenies of consanguineous unions are at increased risk compared to the general population for inheriting recessive disorders, due to the risk of being homozygous by descent for a recessive disease-causing allele. We report on a consanguineous family in which two distinct autosomal recessive conditions segregated, one involving visual impairment that was initially believed to be Leber congenital amaurosis, and the other, congenital alopecia. Identification of the two genetic conditions needed extensive clinical follow-up and was only successful after whole exome sequencing (WES).

In early childhood, Leber congenital amaurosis and achromatopsia are two types of congenital retinal dystrophies that overlap clinically, both presenting with visual impairment and nystagmus [3]. Achromatopsia is characterized by deficient color vision, low visual acuity, photophobia, and nystagmus. In complete achromatopsia there is complete lack of function of all of the three types of retinal cones, responsible for blue, green, and red color vision. Thus, the color vision is absent and rods only mediate visual impulses. In incomplete achromatopsia one or two cone types have residual function, allowing partial color vision [4]. Achromatopsia is an autosomal recessive disorder resulting from dysfunction of one of six genes: *CNGB3*, *CNGA3*, *GNAT2*, *PDE6C*, *PDE6H* [4], and *ATF6* [5]. Achieving an accurate diagnosis in children with retinal dystrophies is important and usually involves genetic testing.

Autosomal recessive forms of alopecia are caused by variants in six genes: *HR*, *DSG4*, *LIPH*, *DSC3*, *DSP,* and *LPAR6* [6]. Variants in *LPAR6* cause hypotrichosis type 8 (OMIM 278150), a type of non-syndromic alopecia, with or without woolly hair and diffuse progressive hair loss, usually beginning in early childhood [7,8].

We report here on a consanguineous Pakistani family in which two rare variants, *PDE6H* and *LPAR6*, are segregated.

## 2. Experimental Section

### 2.1. Clinical Presentation

**Figure 1:**
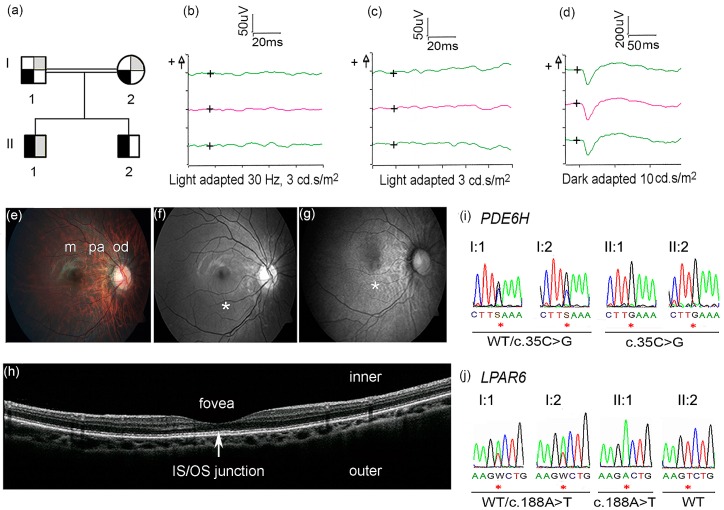
(**a**) Family pedigree showing segregation of incomplete achromatopsia (IA) and alopecia. Black symbol refers to IA, grey symbol refers to alopecia; ¼ filled symbols = carrier, ½ filled symbols = affected. The genotype of the *PDE6H* and *LPAR6* are the following: I:1 WT/c.35C>G; WT/c.188A>T; I:2 WT/c.35C>G; WT/c.188A>T; II:1 c.35C>G/c.35C>G; c.188A>T/ c.188A>T; II:2 c.35C>G/c.35C>G; WT/WT. Ophthalmological examinations (b–h) were performed in the right eye of Patient II:1. (b–d) ERG *x*-axis = time in milliseconds (ms), *y*-axis = response in microvolt (uV); the “+” symbol (on the left side of *y*-axis) indicate positive voltage; the “+” symbols inside the ERG graphs indicate the origin of the axes; red lines indicate median values of two measurements (green lines); (**b**) Cone ERG showed almost absent flicker response at 30 Hz; (**c**) Cone ERG showed almost absent single flash response; (**d**) Dark adapted ERG showed normal rod response; (**e**) Color fundus photography showed normal macula (m) and mild to moderate myopic changes in the peripapillary area (pa) and in the optic disc (od); (**f**) Red-free fundus photography showed normal macula (*); (**g**) Fundus autofluorescence images showed mottling in the macula (*); (**h**) Optical coherence tomography showed normal distribution of retinal layers, without disruption of the inner segment/outer segment junction (arrow); (**i**) Sanger sequencing showed homozygosity for *PDE6H* c.35C>G in Patient II:1 and II:2 and heterozygosity in I:1 and I:2; (**j**) Sanger sequencing showed homozygosity for *LPAR6* c.188A>T in Patient II:1, heterozygosity in I:1 and I:2, and wild-type (WT) sequence in Patient II:2 (the reverse sequence is shown in the figure).

A 15-year-old boy (Figure 1a, II:1) was the first child of first-degree cousins from Pakistan. He was treated for neonatal hyperbilirubinemia; the perinatal period was otherwise normal. Visual contact, however, was first established at 10 weeks (normal <6 weeks). From four months he was observed to develop horizontal nystagmus with involuntary rhythmic horizontal head movements, suggestive of spasmus nutans [9]. He had strabismus and the quality of visual contact was questioned. Spasmus nutans can overlap with the more severe neurological condition of opsoclonus-myoclonus syndrome [10], but follow-up examinations with cerebral MRI, EEG, and screening for malignancy gave normal results. General psychomotor development was normal. Myopia (10 diopters) and astigmatism were diagnosed at eight months. At 11 months, the involuntary head movements had decreased, although a fine nystagmus with small amplitude and high frequency persisted. A precise diagnosis was still missing and at 2.5 years his condition was changed to being loosely labeled “congenital nystagmus with myopia”, complicated by a hitherto unresolved deficiency of hair growth. Since the shaving of his scalp in the neonatal period, his hair had never regrown leaving him bald-headed.

Deficient red-green color vision and profound weak-sightedness were disclosed at nine years, but blue-yellow color vision was normal (Hardy, Rand, Rittler Color Vision Test and HRR Pseudoisochromatic Plate Test, 4th ed., Richmond Products, Inc., Albuquerque, NM, USA). Photophobia was not present. The results from repeated follow-up eye examinations are summarized in Table 1. A clinical diagnosis of Leber congenital amaurosis was eventually proposed. An electroretinogram (ERG) was performed at 11 and 15 years with the patient awake using an Espion E3 Electroretinography System (Diagnosys LLC, Littleton, MA, USA) in accordance with the International Society for Clinical Electrophysiology of Vision (iscev.org). The cone ERG showed an almost absent flicker response at 30 Hz (Figure 1b) and almost absent single flash response (Figure 1c). Importantly, scotopic responses were normal (Figure 1d) and, therefore, the diagnosis of Leber congenital amaurosis was discarded [11]. Color fundus photography showed mild to moderate myopic changes of the optic disc and peripapillary area (Figure 1e). Red-free fundus photography showed normal macula (Figure 1f). Fundus autofluorescence photography showed a mottled pattern in the macula (Figure 1g). The peripheral retina was normal. Optical coherence tomography at age 15 showed a normal foveal pit and normal distribution of retinal layers, without signs of disruption of the inner segment/outer segment junction (Figure 1h).

The patient was referred to one of the authors (PS) for assessment of a possible syndrome comprising the combination of ocular and hair abnormalities. When dissecting the expanded family history, it became clear that these two features segregated as separate inherited phenotypes, as the mother’s sister’s two daughters had alopecia, without visual impairment. It was, therefore, concluded that the patient had inherited two autosomal recessive conditions.

The younger brother, a 10-year-old boy (Figure 1a, II:2) presented at 18 months with myopia (eight diopters), horizontal nystagmus of the left eye, and strabismus treated with Botulinum toxin injections over the years. Examination showed mild to moderate myopic changes of the optic disc and peripapillary area. Discreet, small disruptions of the retinal pigment epithelium in the central macula, only visible by ophthalmoscopy, were observed. At nine years, an ERG, with the patient awake, showed almost absent photopic responses and normal scotopic responses. There was no deterioration in visual acuity over the years (Table 1). At 10 years nystagmus was no longer detectable. His ophthalmological findings were similar to those in Patient II:1. He did not have alopecia.

### 2.2. Materials and Methods

The study was conducted in accordance with the Declaration of Helsinki, and the Regional Ethical Committee approved the research project (REK 2010/1152-1). The parents of the patients signed a written informed consent for the genetic analyses and for publishing the results and photos of the patients.

#### Whole Exome Sequencing (WES) and Data Analysis

DNA from peripheral leukocytes from Patient II:1 and his parents (I:1, I:2) was sheared using a Covaris sonicator (Covaris, Woburn, MA, USA) to produce fragments with an average size of 200 bp. Paired-end Illumina adapters (Illumina, Inc., San Diego, CA, USA) were ligated to the fragments according to the manufacturer’s recommendations. Exome capture was performed with the SureSelect Human All Exon kit v5 (Agilent Technologies, Santa Clara, CA, USA). The exome-captured library was sequenced on an Illumina HiSeq2000 with 100 bp paired end reads. Reads that did not pass Illumina’s standard filter were removed prior to alignment. The remaining reads were aligned to the reference human genome (GRCh37/hg19), using the Burrows-Wheeler aligner tool [12]. PCR duplicates were identified using Picard (broadinstitute.github.io/picard). Approximately 95% of the reads mapped uniquely to the reference sequence yielding an average of 125× coverage per targeted base. Joint variant calling was performed using the Unified Genotyper in the Genome Analysis Tool kit (GATK 2.5-2) [13]. Variants were annotated with snpEff [14] and Variant Effect Predictor using annotations GRCh 37.64 and Ensembl 71, respectively. The Integrative Genomic Viewer [15] was used for data visualization.

The annotated variant files were filtered and analyzed using the software FILTUS 0.99-91 [16]. Variants in genes known to generate false positive signals in exome sequencing were discarded [17], together with variants with minor allele frequency higher than 0.01 according to the 1000 Genome Project [18], the Exome Variant Server (evs.gs.washington.edu/EVS) and an in-house database of 443 exomes of mixed ethnicity. We focused on variants resulting in missense, nonsense, frameshift, and small insertion/deletions in order to find causative variants. Allele frequency was also assessed in the Exome Aggregate database (ExAc, exac.broadinstitute.org), which contains data from more than 60,000 human exomes. Finally, we used the autozygosity functionality of FILTUS to detect regions of homozygosity (ROH) in the patients’ genome. The lengths of identified ROHs were reported in megabases and in centimorgan (according to the Decode recombination map of the human genome [19]).

## 3. Results

Based on the family pedigree (Figure 1a), WES data from Patient II:1 and the parents were analyzed according to a recessive mode of inheritance (Table S1). Variants in 23 genes were detected after data filtering (Table S2). Homozygous variants identified in *PDE6H* (NM_006205.2, OMIM 601190) c.35C>G, p.Ser12* and *LPAR6* (NM_005767.5, OMIM 609239) c.188A>T, and p.Asp63Val (Figure 1i–j)) were considered to be likely disease-causing because of their established involvement in incomplete achromatopsia and alopecia, respectively [8,20,21,22,23]. These variants were validated by Sanger sequencing. We proved the heterozygosity of the parents and homozygosity of Patient II:1 for both variants, whereas Patient II:2 was homozygous for *PDE6H* c.35C>G and wild-type for *LPAR6* c.188A (Figure 1i–j).

**Figure 2:**
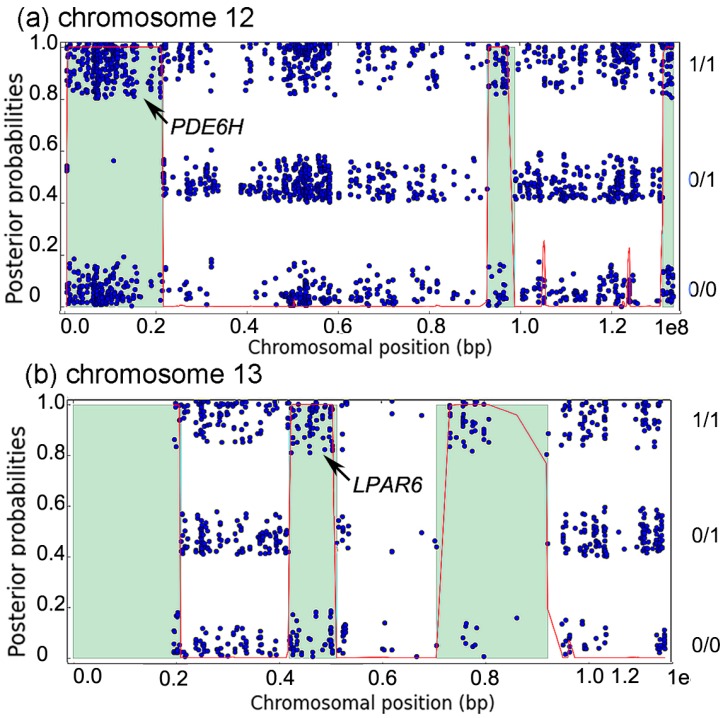
(**a**,**b**) Autozygosity analysis of chromosomes 12 and 13 in patient II:1. Each blue dot represents a variant. It is placed in the lower band if homozygous for the reference allele (0/0), in the middle band if heterozygous (0/1), and in the top band if homozygous for a non-reference allele (1/1). The red curve shows posterior probability of autozygosity, and predicted autozygous regions are colored in light green. The autozygous regions containing *PDE6H* (chromosome 12) and *LPAR6* (chromosome 13) are indicated by the arrows.

Autozygosity analysis of the patient revealed that both *PDE6H* and *LPAR6* were surrounded by regions of homozygosity (ROH), of lengths 21 Mb (34 cM) and 9 Mb (10 cM), respectively (Figure 2a,b). Such ROH sizes are typical for autozygous segments resulting from first cousin marriage, and suggest that both *PDE6H*:c.35C>G and *LPAR6*:c.188A>T are in an autozygous state in the patient, inherited via both parents from one of their shared grandparents.

In the ExAc database (exac.broadinstitute.org), containing more than 60,000 human exomes data), the *PDE6H* c.35C>G variant has been identified in heterozygosity in nine out of 61,228 individuals (seven European non-Finnish, one Finnish, one African), but in none of the 16,590 alleles of South Asian individuals. The *LPAR6* c.188A>T variant is not present in the ExAc database. Sanger sequencing of 180 in-house Pakistani controls did not reveal the *PDE6H* c.35C>G variant. The Pakistani controls were not analyzed for *LPAR6* c.188A>T, because the variant was reported among Pakistani [8,20,21,23].

The DNA variants identified in the study have now been reported to the ClinVar database (ncbi.nlm.nih.gov/clinvar/): SCV000256082 *PDE6H* c.35C>G, p.Ser12*; SCV000256083 *LPAR6* c.188A>T, p.Asp63Val.

## 4. Discussion

For the proband reported here we describe the co-existence of a rare ophthalmological diagnosis eventually pinpointed as incomplete achromatopsia and congenital alopecia, and incomplete achromatopsia in a younger brother. The genetic variants causing the two separate clinical phenotypes were documented using WES.

It was not straightforward to establish the correct ophthalmological diagnosis. Patient II:1 was initially suspected of having spasmus nutans bordering opsoclonus-myoclonus, before being labeled “congenital nystagmus with myopia”, and was subsequently thought to have Leber congenital amaurosis, carrying a more pessimistic prognosis than achromatopsia. Congenital hypotrichosis with juvenile macular dystrophy (HJMD, OMIM 601553), due to biallelic variants in *CDH3* [24], manifests with both features present in Patient 1, the alopecia and the ophthalmological disease. However, HJMD was ruled out in Patient 1 because, from the family history, it was evident that the two conditions segregated independently. WES eventually revealed the true cause of the retinal dystrophy.

This is the second confirmatory report of a *PDE6H* variant causing incomplete achromatopsia. Interestingly, in both reports the variant is c.35C>G, p.Ser12*, but it arises in different ethnicities: South Asian in our family and Caucasian in the first study, which describes three patients from two families, sharing a common haplotype around the variant [22].

*PDE6H* encodes the γ subunit (PDEγ) of the cyclic guanosine monophosphate (cGMP) cone phosphodiesterase (PDE) enzyme, essential for the phototransduction [25]. The c.35C>G, p.12Ser* nonsense variant causes lack of functional PDEγ, affecting the cone PDE in the phototransduction, possibly leaving cGMP-gated channels permanently closed [22].

Immunohistochemistry on mouse retinal sections using a PDE6H antibody stained all cones [26]. However, patients with *PDE6H* c.35C>G have preserved S-cone function and disturbed M- and L-cone functions (Table 1) [22]. The preserved S-cone function in the absence of PDEγ suggests possible compensation for the loss of PDE6H in the PDE. This was demonstrated in *PDE6H* knockout mice, in which no photoreceptor dysfunction was recorded in the absence of PDE6H, due to functional complementation by the rod PDE6G [26].

Incomplete achromatopsia caused by defective PDE6H presents a rather homogeneous ophthalmological profile [22]. The two siblings in this report, and the three patients previously-described homozygous for *PDE6H* c.35C>G and incomplete achromatopsia, presented in early childhood with strong red-green color vision deficiency and normal blue-yellow color vision (Table 1). On ERG they all had normal scotopic, but severely reduced photopic, signals. Visual acuity was persistently poor, but did not deteriorate.

Approximately 60% of marriages in Pakistan are estimated to be consanguineous [27] and expose the progeny to higher risk of homozygosity by descent for a recessive disease-causing allele. In this Pakistani family Patient II:1 had two autosomal recessive conditions: incomplete achromatopsia and alopecia (Figure S1).

LPAR6 is a G protein coupled receptor for lysophosphatidic acid (LPA), expressed in the inner root sheath of the hair follicle. Molecular modeling indicated that p.Asp63Val in the transmembrane helices 2 domain alters LPAR6 conformation and inhibits its binding to LPA, a key factor for inner root sheath differentiation and hair development [23]. Individuals with the *LPAR6* c.188A>T, p.Asp63Val had a variable degree of alopecia, some with predominantly woolly hair and some with only sparse hair [8,20,21,23]. Interestingly, the variant has only been identified within the Pakistani population [7,8,23] but is not reported in the ExAc database, suggesting that it is not distributed too widely within the Pakistani population in which it may have originated.

## 5. Conclusions

This study illustrates the dilemma faced when determining how to best distinguish amongst rare congenital retinal dystrophies. Mode of inheritance and clinical evaluation over time supplemented with ERG examination will allow the ophthalmologist to rule out some diseases, but timely establishment of the exact diagnosis requires genetic testing.

## Figures and Tables

**Table 1:** Comparison of the ophthalmological presentation of the five patients reported with biallelic c.35C>G variant in *PDE6H*.

Examination	This Report		Kohl et al. [22]		
	Patient II:1	Patient II:2	NL-II:1	BE-II:1	BE-II:2
Age last visit	15 years	10 years	45 years	22 years	20 years
Best corrected visual acuity	OD 6/15; OS 6/24	OD 6/20; OS 6/15	OD 20/125; OS 20/125	OD 20/63; OS 20/63	OD 20/200; OS 20/100
Refraction	OD −10.5, −2.5 × 180; OS −10.5, −2.5 × 160	OD −9.0, −2.0 × 30; OS −9.5, −2.5 × 160	OD −7.5; OS −6.5	OD −13.5; OS −14.25	OD −8.25; OS −8.25
Nystagmus	Present	Present *	Present	Absent	Absent
Photophobia	Absent	Absent	Present	Present	Present
Goldmann perimetry	Normal	NA	NA	Normal	Normal
Fundoscopy	Changes in ODi and PA.	Changes in ODi and PA. Discreet, small disruptions of RPE in central fovea	Normal	Normal color of ODi, large temporal myopic crescents	Normal color of ODi, large temporal myopic crescents
Retina	Mild myopic changes	Mild myopic changes	NA	Irregular atrophic depigmentation	Irregular atrophic depigmentation
Macular autofluorescence	Mottled	Mottled	NA	NA	NA
**Color vision**					
Red-green deficiency	Severe	Moderate	Severe	Moderate	Moderate
Blue-yellow	Normal	Normal	Normal	Normal	Normal
**ERG**					
Rods	Normal	Normal	Normal	Normal	Normal
Cones single flash	Weak	Weak	Absent	Absent	Absent
Cones 30 Hz flicker	Weak	Weak	Absent	Absent	Absent
**OCT**					
IS/OS junction	Normal	Normal	NA	Disrupted	Disrupted
Fovea	Normal	Normal	NA	NA	NA

Legend: ERG—Electroretinography; IS/OS—Inner segment/Outer segment; NA—Not available; PA—Peripapillary area; OCT—Optical coherence tomography; ODi—Optic disc, OD (Oculus dexter)—right eye; OS (Oculus sinister)—left eye; RPE—Retinal pigment epithelium; * Present only transiently.

## Supplementary Materials

The following are available online at www.mdpi.com/2073-4425/7/8/41/s1, Figure S1: Patient 1 at the age of 13, alopecia is evident, Table S1: Analysis of the WES data of Patient II:1 and his parents, according to a recessive mode of inheritance (variants after filtering are listed), Table S2: Number of genetic variants obtained after WES data filtering
